# The role of experience in parenting beliefs of British and Italian women during pregnancy

**DOI:** 10.1002/imhj.22014

**Published:** 2022-10-11

**Authors:** Eleonora Mascheroni, Maria Grassi, Andrea Bonanomi, Rebecca Sperotto, Sita Deeg, San Hung, Ruixue Xia, Chiara Ionio, Terry Kit‐fong Au, Merideth Gattis

**Affiliations:** ^1^ School of Psychology Cardiff University Cardiff UK; ^2^ 0–3 Center for the at‐Risk Infant Scientific Institute IRCCS Eugenio Medea Bosisio Parini Italy; ^3^ Department of Psychology Centro di Ricerca sulle Dinamiche evolutive ed educative (CRIdee) Catholic University of Milan Milan Italy; ^4^ Department of Statistical Science Catholic University of Milan Milan Italy; ^5^ Department of Psychology University of Hong Kong Hong Kong SAR China; ^6^ Department of Psychology Northwest Normal University Lanzhou Gansu China

**Keywords:** attunement, parenting beliefs, pregnancy, structure, transition to parenthood, coordinación armónica, estructura, creencias de crianza, embarazo, paridad, harmonisation, structure, croyances de parentalité, grossesse, parité, Einfühlungsvermögen, Struktur, elterliche Überzeugungen, Schwangerschaft, Parität, 情動調律、育児体制、育児信念、妊娠、平等性, 调谐, 周密安排, 育儿观念, 怀孕, 孕产次数, **الكلمات المفتاحية**: التناغم ، التركيب ، معتقدات التربية ، الحمل ، التكافؤ

## Abstract

To understand the role of experience in parenting beliefs about caring for infants, we examined the parenting beliefs of pregnant women who were expecting their first child with those of pregnant women who already had at least one other child. A culturally diverse sample of 550 British and Italian women completed self‐report measures evaluating their beliefs about the value of attunement and structure in caregiving, parenting self‐efficacy, and home chaos. Psychometric evaluation confirmed the two‐factor structure of the Baby Care Questionnaire (BCQ) for measuring attunement and structure but did not support configural invariance across the different samples. Beliefs about attunement and structure were related to parenting experience: pregnant women who already had at least one other child reported stronger beliefs in attunement, whereas pregnant women expecting their first child reported stronger beliefs in structure. Regression analyses revealed that the associations between parenting beliefs and experience remained when controlling for country, age, and education. Despite the limitations imposed by the lack of configural invariance, this cross‐sectional, cross‐cultural study constitutes an important first step in examining the relations between parenting experience and parenting beliefs during pregnancy.

## Resumen

Para comprender el papel de la experiencia en las creencias de crianza sobre el cuidado de los infantes, examinamos las creencias de crianza de mujeres embarazadas que esperaban su primer niño con aquellas de mujeres embarazadas que ya tenían por lo menos otro niño. Un grupo muestra culturalmente diverso de quinientas cincuenta mujeres británicas e italianas completó medidas auto reportadas de evaluación de sus creencias acerca del valor de la coordinación armónica y la estructura en cuanto al cuidado, la auto efectividad de la crianza, así como el caos en casa. La evaluación sicométrica confirmó la estructura de dos factores del Cuestionario de Cuidado del Bebé (BCQ: Winstanley y Gattis, 2013) para medir la coordinación armónica y la estructura, pero no apoyó la invariabilidad configuracional a través de los diferentes grupos muestra. Las creencias acerca de la coordinación armónica y la estructura se relacionaron con la experiencia de la crianza: las mujeres embarazadas que ya tenían por lo menos otro niño reportaron creencias más fuertes en cuanto a la coordinación armónica, mientras que las mujeres embarazadas que esperaban su primer niño reportaron creencias más fuertes en cuanto a la estructura. Los análisis de regresión revelaron que las asociaciones entre las creencias de crianza y la experiencia se mantenían siendo las mismas cuando se usaron los controles referentes a país, edad y educación. A pesar de las limitaciones impuestas por la falta de la invariabilidad configuracional, este estudio interseccional constituye un importante paso en el examen de las relaciones entre la experiencia de crianza y las creencias sobre la crianza durante el embarazo.

## Résumé

Afin de comprendre le rôle de l'expérience dans les croyances de parentalité sur la manière de prendre soin des bébés, nous avons examiné les croyances de parentalité de femmes enceintes qui attendaient leur premier enfant avec celles de femmes enceintes ayant déjà eu au moins un enfant. Un échantillon culturellement divers de cinq cent cinquante femmes britanniques et italiennes ont rempli des mesures auto‐rapportées évaluant leurs croyances concernant la valeur de l'harmonisation et de la structure dans les soins à l'enfant, l'auto‐efficacité de parentalité, et le chaos à domicile. L’évaluation psychométrique a confirmé la structure à deux facteurs du Questionnaire du Soin au Bébé (abrégé BCQ en anglais; Winstanley & Gattis, 2013) pour la mesure de l'harmonisation et de la structure mais n'a pas soutenu l'invariance de configuration au travers des différents échantillons. Les croyances sur l'harmonisation et la structure étaient liées à l'expérience de parentalité: les femmes enceintes qui avaient déjà eu un enfant ont fait état de croyances plus fortes dans l'harmonisation, alors que les femmes enceintes attendant leur premier enfant ont fait état de croyances plus fortes dans la structure.

Des analyses de régression ont révélé que les liens entre les croyances de parentalité et l'expérience demeuraient quand on contrôlait pour le pays, l’âge et l’éducation. En dépit des limitations imposées par le manque d'invariance de configuration, cette étude transversale et multiculturelle constitue une étape importante dans l'examen des relations entre l'expérience de parentalité et les croyances de parentalité durant la grossesse.

## Zusammenfassung

Die Rolle der Erfahrung bei elterlichen Überzeugungen britischer und italienischer Frauen während der Schwangerschaft

Um die Rolle der Erfahrung bei elterlichen Überzeugungen bezüglich der Betreuung von Säuglingen zu verstehen, verglichen wir elterliche Überzeugungen von schwangeren Frauen, die ihr erstes Kind erwarteten, mit denen von schwangeren Frauen, die bereits mindestens ein weiteres Kind hatten. Eine kulturell gemischte Stichprobe von fünfhundertfünfzig britischen und italienischen Frauen füllte Selbstauskunftsbögen aus, in denen ihre Überzeugungen bezüglich des Werts von Einfühlungsvermögen und Struktur in der Betreuung, elterliche Selbstwirksamkeit und häusliches Chaos erhoben wurden. Die psychometrische Auswertung bestätigte die Zwei‐Faktoren‐Struktur des Baby Care Questionnaire (BCQ; Winstan‐ley & Gattis, 2013) für die Messung von Einfühlungsvermögen und Struktur, konnte aber keine Konfigurationsinvarianz über die verschiedenen Stichproben hinweg belegen. Die Überzeugungen bezüglich Einfühlungsvermögen und Struktur hingen mit der Erfahrung als Elternteil zusammen: Schwangere Frauen, die bereits mindestens ein weiters Kind hatten, gaben stärkere Überzeugungen in Bezug auf Einfühlungsvermögen an, während schwangere Frauen, die ihr erstes Kind erwarteten, stärkere Überzeugungen in Bezug auf Struktur angaben. Regressionsanalysen ergaben, dass die Zusammenhänge zwischen elterlichen Überzeugungen und Erfahrung auch dann bestehen blieben, wenn für das Herkunftsland, das Alter und die Bildung kontrolliert wurde. Trotz der Einschränkungen, die sich aus der fehlenden Konfigurationsinvarianz ergeben, stellt diese kulturübergreifende Querschnittsstudie einen wichtigen ersten Schritt bei der Untersuchung der Zusammenhänge zwischen elterlicher Erfahrung und elterlichen Überzeugungen während der Schwangerschaft dar.

## 抄録

妊娠中のイギリス人女性およびイタリア人女性の育児信念における経験の役割

乳幼児の世話に関する育児信念における経験の役割を理解するために、第一子を妊娠中の妊婦と、他に少なくとも一人の子どもを持つ妊婦の育児信念を検討した。文化的に多様なイギリス人とイタリア人の女性550名が、育児における情動調律と育児体制の価値、子育ての自己効力感、家庭の混乱に関する信念を評価する自記式測定法に回答した。心理測定評価では、Baby Care Questionnaire (BCQ; Winstanley & Gattis, 2013) の情動調律と育児体制を測定する2因子構造を確認したが、異なるサンプル間での配置不変性は支持されなかった。情動調律と育児体制に関する信念は、子育て経験と関連していた。少なくとも他に1人の子どもを持つ妊婦は、情動調律に関する信念が強く、一方、第一子を妊娠している妊婦は育児体制に関する信念が強いと報告された。回帰分析の結果、国、年齢、教育でコントロールしても、子育て信念と経験との関連は保たれていた。配置不変性の欠如による限界はあるものの、この横断的・異文化間研究は、妊娠中の育児経験と育児信念の関係を検討する上で重要な第一歩を構成するものである。

## 摘要

为了了解经验在育儿观念中的作用, 我们研究了怀有第一个孩子的孕妇和已经有至少一个孩子的孕妇的育儿观念。来自不同文化背景的550名英国和意大利女性完成了自我报告测量, 以评估她们对护理、育儿自我效能和家庭混乱中使用调谐及周密安排的价值观。心理测量评估证实了“婴儿护理问卷” (BCQ; Winstanley & Gattis, 2013) 用于测量调谐和周密安排的双因素结构, 但不支持不同样本的构形不变性。关于调谐和周密安排的观念与育儿经验有关:已经至少有一个孩子的孕妇报告了更强的调谐观念, 而怀有第一个孩子的孕妇报告了更强的周密安排观念。回归分析显示, 当国家、年龄和教育程度作为控制条件后, 育儿观念和经验之间的关联仍然存在。尽管由于缺乏构形不变性而受到限制, 但这项跨种群、跨文化的研究是检验怀孕期间育儿经验和育儿观念之间关系的重要第一步。

## ملخص

دور الخبرة في معتقدات التربية الوالدية عند المرأة البريطانية والإيطالية أثناء الحمل

لفهم دور الخبرة في معتقدات التربية حول رعاية الأطفال ، قمنا بدراسة المعتقدات الرعاية الوالدية عند النساء الحوامل اللواتي كن يتوقعن أول طفل لهن ومقارنتها بالمعتقدات الخاصة بالنساء الحوامل اللواتي أنجبن بالفعل طفلًا واحدًا آخر على الأقل. أكملت عينة متنوعة ثقافيًا من خمسمائة وخمسين امرأة بريطانية وإيطالية مقاييس التقرير الذاتي لتقييم معتقداتهن حول أهمية التناغم ووجود بنية محددة لتقديم الرعاية ، والكفاءة الذاتية في الرعاية الوالدية ، والفوضى المنزلية. أكد التقييم السيكوميتري البنية ذات العاملين لاستبيان رعاية الطفل (BCQ ؛ Winstanley & Gattis ، 2013) لقياس التناغم والهيكل ، لكنه لم يدعم الثبات الهيكلي عبر العينات المختلفة. كانت المعتقدات حول التناغم والبنية مرتبطة بتجربة الأبوة والأمومة: أبلغت النساء الحوامل اللائي لديهن بالفعل طفل واحد آخر على الأقل عن معتقدات أقوى في التناغم والتعديل ، بينما أبلغت النساء الحوامل اللائي يتوقعن طفلهن الأول عن معتقدات أقوى في البنية. كشفت تحليلات الانحدار أن الارتباطات بين معتقدات التربية والخبرة بقيت واضحة عند التحكم في البلد والعمر والتعليم. وعلى الرغم من القيود التي يفرضها الافتقار إلى الثبات التركيبي ، فإن هذه الدراسة المقطعية متعددة الثقافات تشكل خطوة أولى مهمة في دراسة العلاقات بين تجربة الأبوة والأمومة ومعتقدات التربية أثناء الحمل.

## 1 INTRODUCTION

Parenting beliefs are cognitions about how to care for children and expectations about their development (Bugental & Johnston, 2000; Goodnow, 1988; Miller, 1988; Schaefer & Bell, 1958). Although parenting beliefs have great theoretical and practical significance, we still know relatively little about how parenting beliefs are formed, and in particular the role of parenting experience in beliefs (Palkovitz & Copes, 1988; Ryan & Padilla, 2019). Research examining the role of experience in parenting beliefs during pregnancy has the potential to contribute to scientific understanding of the development of parenting beliefs and, importantly, how practitioners can best support first‐time parents making the transition to parenthood.

## 2 PARENTING BELIEFS ABOUT CAREGIVING DURING INFANCY

Parenting beliefs have been widely studied as a potential factor in caregiving during infancy, especially in relation to sleeping, feeding, and crying problems (e.g., Hughes et al., 2012; Tikotzky & Sadeh, 2009). For example, parents differ in their beliefs about whether waking in the night indicates infant needs, as well as beliefs about how much parents versus infants are responsible for the transition to sleep (Galbally et al., 2018; Morrell, 1999; Sadeh et al., 2007). Furthermore, in at least some cultural contexts, beliefs about parental responsibility for the transition to sleep are positively associated with infant sleeping problems (Galbally et al., 2018; Morrell, 1999; Sadeh et al., 2007). Parent‐centered cognitions are also associated with feeding and crying problems (Haltigan et al., 2012; Jansen et al., 2015; Leerkes et al., 2010; Thompson et al., 2009). In contrast, child‐centered parenting beliefs may encourage attention to infant cues and autonomy granting, and as a result lead to better outcomes for children (e.g., Thompson et al., 2009). Importantly however, nearly all studies of parenting beliefs about caregiving during infancy have been studied postpartum, or in other words, in experienced parents only. As a result, it is difficult to establish whether associations between parenting beliefs and child outcomes reflect causality, and if so, in what direction.

Winstanley and Gattis (2013) proposed that two beliefs guide caregiving during infancy across the contexts of sleeping, feeding, and soothing. Attunement refers to beliefs about the value and utility of infant cues signaling hunger and satiety, drowsiness and wakefulness, distress and soothing, and parental responding to those cues. Structure refers to beliefs about the value of regularity and routines in infant care, such as following a schedule in day‐to‐day caregiving. Attunement and structure are not conceptualized as opposite or mutually exclusive caregiving types, but instead as orthogonal dimensions along which parenting beliefs might vary independently (Winstanley & Gattis, 2013). The distinction between attunement and structure does not neatly correspond to the distinction between child‐ versus parent‐centered cognitions discussed above. Rather, both attunement and structure are beliefs that can support caregiving decisions and positive outcomes for children: attunement through attention to infant cues, and structure through attention to regularity and routines that can help infants predict their environment.

Winstanley and Gattis (2013) designed and evaluated the Baby Care Questionnaire (BCQ) to measure attunement and structure beliefs, as well as parenting behaviors such as breastfeeding and bed‐sharing. To measure beliefs, the BCQ asks parents to rate their agreement versus disagreement with statements about infant sleeping, feeding, and soothing such as “*Babies benefit from physical contact with parent(s) when they wake during the night”* (attunement) and “*It is important to introduce a sleeping schedule as early as possible”* (structure). In a study with experienced parents, beliefs about attunement were positively associated with proximal parenting behaviors such as bed‐sharing, breastfeeding, and holding infants, while beliefs about structure were negatively associated with breastfeeding (Winstanley & Gattis, 2013). In addition, attunement and structure interacted to predict infant crying: parents with strong beliefs in attunement and weak beliefs in structure reported that their infants cried more. Winstanley and Gattis’ (2013) findings provide some empirical evidence that attunement and structure describe parenting beliefs that are coherent across different contexts of caregiving and are associated with positive parenting behaviors and child outcomes.

## 3 THE ROLE OF EXPERIENCE IN PARENTING BELIEFS

Although parenting experience is generally thought to shape parenting beliefs and behavior, empirical evidence is limited (Rubin & Chung, 2006; Sigel & McGillicuddy‐DeLisi, 2002; Valsiner & Lightfoot, 1987; Weisner et al., 1977). Some studies have examined how experiences of care *during childhood* influence later parenting beliefs, but little is known about how parents’ experience *as parents* influences their parenting beliefs (Barrett & Fleming, 2011; Belsky, 1984; Daggett et al., 2000). Both intuition and evidence, however, suggest that experience matters. Parents perceive infant cries as less aversive and distressing than non‐parents do, but respond to infant crying more quickly (Irwin, 2003; Lester & LaGasse, 2008; Sadeh et al., 2016; Tikotzky & Sadeh, 2009; Zeskind & Lester, 1978). Mothers of two or more children have more parenting confidence compared to first‐time mothers (Hsu & Lavelli, 2005; Porter & Hsu, 2003; Salonen et al., 2009). In addition, parents’ beliefs about nurturing and responding to infants become stronger during the months following the birth of their first child, whereas beliefs about restrictiveness, authoritarianism, and limit‐setting become weaker (Kahn et al., 2018; Scott & Hill, 2001; Tikotzsky & Sadeh, 2009).

Importantly, however, studies investigating parenting beliefs about caregiving during infancy have for the most part been conducted postpartum with parents already engaged in the task of parenting. One reason for the paucity of research on parenting beliefs amongst pregnant women is that most measures of parenting beliefs were designed for experienced parents (Haltigan et al., 2012; Morrell, 1999; Thompson et al., 2009). For example, the Maternal Cognitions about Infant Sleep Questionnaire measures parenting beliefs about sleep by asking respondents to rate statements such as “*It is alright to let my child cry at night*” with respect to their own infant.

Because the BCQ measures beliefs by asking parents to rate statements about infants in general, rather than their own infant, it is a good candidate instrument for investigating the measurement of parenting beliefs during pregnancy. Winstanley and Gattis (2013) reported cross‐sectional data indicating that pregnant women and current parents had similar parenting beliefs about attunement and structure, but the proportion of pregnant women in their study was small, and their analyzes did not distinguish between pregnant women expecting their first child and pregnant women who already had at least one child. Winstanley et al. (2014) evaluated parenting beliefs at birth and again 5 months later: attunement beliefs were stable from birth to five months for parents of both full‐term and preterm infants whereas structure beliefs were stable for parents of full‐term but not preterm infants. To examine the role of parenting experience in parenting beliefs, we decided to use the BCQ to investigate parenting beliefs in pregnant women, and to distinguish between women expecting their first child and women who already had one or more children, and thus had parenting experience.

## 4 PARENTING BELIEFS IN CULTURAL AND SOCIETAL CONTEXT

Although the primary tasks of caregiving during infancy are universal, specific parenting behaviors, such as where infants sleep and whether they have regular bedtimes and routines, do vary across cultural contexts (Mindell et al., 2010; Morelli et al., 1992). Many studies have uncovered both universals and variation in infant care across cultures (e.g., Bornstein et al., 2017; Lozoff & Brittenham, 1979; van Sleuwen et al., 2007). Parenting beliefs are important to understanding parenting behaviors in different cultural contexts. For example, Morelli et al. (1992) found that American infants generally slept in separate beds from their parents, and often separate rooms, whereas Guatemalan infants slept in the parents’ bed. Furthermore, American parents described their sleeping arrangements as helping infants develop independence, whereas Guatemalan parents described their sleeping arrangements as promoting closeness. Thus, not only specific parenting beliefs but also more broadly inter‐related beliefs including parenting goals, practices, and child outcomes may cohere and vary in character as well as frequency across cultural contexts (Prevoo & Tamis‐LeMonda, 2017).

Because cross‐cultural evidence is crucial to properly characterizing parenting beliefs, we collected data including a culturally diverse sample of and British and Italian women. Previous research has documented both similarities and differences in parenting behavior in the two countries (e.g., Bozicevic et al., 2021), and there is limited evidence comparing parenting beliefs across the two countries. We did not have a priori hypotheses about potential cross‐cultural differences in parenting beliefs about attunement and structure. Based on previous findings of relations between parent age and education and parenting beliefs, and because in some cases, ostensible differences between cultures are better explained by other variables such as education and age, we also included these factors (Willemsen & van de Vijver, 1997).

Our study also included two further contextual factors to help us evaluate the validity of the BCQ: parenting self‐efficacy and chaotic home environments (Winstanley & Gattis, 2013; Winstanley et al., 2014). Parenting self‐efficacy refers a parent's belief in their ability to successfully perform the nurturing role (Coleman & Karraker, 1997). Because a positive relation has been reported between parenting self‐efficacy and maternal sensitivity and responsiveness, we predicted a positive relation between parenting self‐efficacy and attunement (Stifter & Bono, 1998; Teti & Gelfand, 1991; Tucker et al., 1998). Chaotic home environments are defined as household settings that exhibit disorganization, uncertainty, and a lack of structure and routines (Corapci & Wachs, 2002; Wachs & Evans, 2010). We used the Confusion, Hubbub, and Order Scale (CHAOS; Matheny et al., 1995) to measure of chaotic home environments, and predicted a negative relation between structure as measured by the BCQ and CHAOS scores.

## 5 OUR STUDY

The aim of our study was to evaluate the role of parenting experience in parenting beliefs during pregnancy. To achieve this overall aim we defined two objectives.

Our first objective was to further evaluate the psychometric properties of the BCQ. Although Winstanley et al. (2013, 2014) reported psychometric evaluations of the BCQ, psychometric evaluation and construct validity are continuous processes that benefit from further testing with new populations and new tests. Furthermore, pregnant women were only a small proportion of their overall sample. We tested the two‐factor structure of the BCQ, evaluated invariance across levels of parenting experience and countries, and evaluated concurrent validity by comparing attunement with parenting self‐efficacy and structure with home chaos.

Our primary objective was to evaluate the role of parenting experience in parenting beliefs during pregnancy. To this end we investigated the relations between parenting experience and beliefs about attunement and structure in caregiving for infants. To evaluate the contribution of experience relative to other factors, we also considered the country where pregnant women lived and their age and education.

## 6 METHODS

### 6.1 Participants

We recruited a sample of Italian and British pregnant women between December 2017 and June 2018 via boosted Facebook posts with a link to our study in Qualtrics. Facebook is widely used in both countries. The two eligibility criteria were: currently pregnant and capable of giving consent (aged 16 or above in the United Kingdom and 18 or above in Italy). All participants provided informed consent. Women who agreed to participate but did not fulfill both eligibility criteria or completed less than 75% of the survey (*n* = 637) were excluded from the final sample. A total of 550 pregnant women (*n* = 290 in Italy; *n* = 260 in United Kingdom) fulfilled all inclusion criteria. Study procedures were reviewed and approved by Cardiff University School of Psychology Research Ethics panel.

### 6.2 Design and procedures

We asked participants to provide information about their age, education, parenting experience, and non‐parental caregiving experience and to complete three self‐report measures. We used the data from this correlational design to evaluate to address the main aim of the current study of investigating the role of parenting experience in parenting beliefs.

### 6.3 Measures

#### 6.3.1 Baby Care Questionnaire (BCQ)

The BCQ (Winstanley & Gattis, 2013) asks parents to rate statements about caregiving in the contexts of sleeping, feeding, and soothing on a 4‐point Likert scale ranging from 1 “*strongly disagree*” to 4 “*strongly agree*.” The statements form two scales: Attunement and Structure. Individual scores for each scale are calculated by averaging across items. Only statements assessing parenting beliefs were included; BCQ items assessing parenting behaviors were not included.

We followed four steps to help us establish a foundation for interpreting similarities and differences in parenting beliefs across populations: evaluation of constructs and items by a multi‐cultural panel; translation and modification of existing items; cognitive interviews with the target populations; and psychometric evaluation (DeVellis, 2016; Erkut, 2010; Gattis et al., 2019; Pena, 2007; Willis & Miller, 2011). In the first step, panel members with expertise in the study of infant development and parenting and with relevant cultural experience and language skills evaluated conceptual and item equivalence, using the BCQ items developed and evaluated by Winstanley and Gattis (2013). Based on advice from Erkut (2010) we also generated and evaluated new items to potentially add to the BCQ. Our initial evaluation indicated that these items did not perform as desired so we did not include them in further analyzes. In the second step, seven items from Winstanley and Gattis (2013) were modified to increase simplicity, clarity, and consistency across languages: “*Babies benefit from physical contact with parent(s) when they wake during the night*” was changed to “*It is important for babies to have physical contact with parents when they wake during the night*”; “*When babies cry in the night to check if someone is near, it is best to leave them”* was changed to “*When babies cry in the night to check if someone is near, it is best to not react”*; “*Babies benefit from a quiet room to sleep”* was changed to “*It is important for babies to sleep in a quiet room”*; “*Babies benefit from a fixed napping/sleeping schedule*” was changed to “*It is important for babies to have a sleeping schedule*”; “*Holding babies frequently during the day makes them more demanding*” was changed to “*The more a baby is held, the more he or she demands attention*”; “*Responding quickly to a crying baby leads to less crying in the long run”* was changed to “*Responding quickly to a crying baby leads to more crying in the long run”*; and “*Leaving a baby to cry can cause emotional insecurity”* was changed to “*Leaving a baby to cry can cause emotional problems”*. All other items from Winstanley and Gattis (2013) were retained unchanged. In the third step, trained researchers conducted cognitive interviews with 5 Italian and 13 British parents (recruited from university‐based volunteer participant pools in each country). Researchers asked parents to read questions aloud and respond to them while describing their thought processes. Panel members analyzed interview transcripts to identify possible problematic and/or unclear items and discussed the items to reach an agreement on item improvements. The final step, psychometric evaluation, is reported in the section on preliminary analyses.

#### 6.3.2 Confusion, Hubbub, and Order Scale (CHAOS)

The CHAOS (Matheny et al., 1995) is a 15‐item self‐report tool designed to measure disorganization, noise, and unpredictability in the home environment. Women rated how well statements described their home environment on a 4‐point Likert scale, ranging from: not at all like your own home (1), a little bit like your own home (2), somewhat like your own home (3), or very much like your own home (4). Seven items assess routines and organization (e.g. “*First thing in the day, we have a regular routine at home*”), and eight items assess disorganization and confusion (e.g., “*You can't hear yourself think in your home*”). Because psychometric analyses by Matheny et al. (1995) indicated that all items form a single factor, we reverse‐scored appropriate items and then calculated a total mean score for each participant. A higher score signified a more chaotic home environment. The CHAOS scale good internal consistency and test‐retest reliability (Matheny et al., 1995), and has been used in pregnant women (Colicchia et al., 2016). Although the CHAOS has been widely used in multiple countries, we were not able to locate an existing version in Italian. We therefore translated the English CHAOS into Italian. We conducted cognitive interviews (*n* = 3) with the Italian CHAOS in advance of our main study. The interviews confirmed that the measure had been translated in a proper way and the questions were interpreted consistently from Italian women. For the current study the CHAOS showed good internal consistency (Cronbach alpha = .83).

#### 6.3.3 Maternal self‐efficacy in nurturing role questionnaire

The Maternal Self‐Efficacy in Nurturing Role Questionnaire (Hsu & Lavelli, 2005; Porter & Hsu, 2003) contains 16 items that assess how participants feel about becoming a parent, for example, “*I feel unprepared in becoming a parent*” and “*I feel I can catch on quickly to the basic skills of caring for my child*.” Participants rated each item as not at all representative of them (1), slightly representative of them (2), moderately representative of them (3) or strongly representative of them (4). We calculated a total maternal efficacy score for each participant by reverse‐scoring appropriate items and then summing across all items. A higher score indicated a higher level of self‐efficacy. We used existing English and Italian versions of the maternal self‐efficacy measure (Hsu & Lavelli, 2005; Porter & Hsu, 2003). For the current study this tool showed good internal consistency (Cronbach alpha = .85).

### 6.4 Data analysis plan

Data were analyzed with IBM Amos 23 software and the IBM SPPS 24 software.

#### 6.4.1 Preliminary analyses

Based on the theoretical framework and subsequent exploratory and confirmatory analyses reported by Winstanley and Gattis (2013), we conducted a Confirmatory Factor Analysis (CFA) on the 30‐item BCQ (13 items for Attunement; 17 items for Structure) (DeVellis, 2016). The model fit resulting from these CFAs was evaluated using the Comparative Fit Index (CFI), the Root Mean Square Error of Approximation (RMSEA), the Akaike Information Criterion (AIC), and the Bayesian Information Criterion (BIC). According to van de Schoot et al. (2012) a CFI > .90 is acceptable and > .95 preferred, while a RMSEA < .08 is acceptable and < .05 preferred. Smaller values of AIC and BIC are preferred. To evaluate the comparability of our target constructs across groups, we tested the invariance of attunement and structure across levels of parenting experience (pregnant women expecting their first child vs. pregnant women who already had at least one child) and across country (UK vs. Italy) (Dimitrov, 2010; Schmitt & Ali, 2015; van de Schoot et al., 2012). We also tested the concurrent validity of attunement and structure: Pearson's correlations were used to examine the relations between self‐efficacy and attunement and between household habits and structure.

#### 6.4.2 Main analyses

Separate ANOVAS for attunement and structure tested differences between groups considered as independent variables: (a) parenting experience (first‐time vs experienced) and (b) country (UK vs. Italy). To further evaluate whether parenting experience predicted beliefs about attunement and structure, regression analyses controlled for country and woman's socio‐demographic characteristics (i.e., age; level of education). We conducted two hierarchical regression models (with either attunement or structure as outcome variable) with the following independent variables: parenting experience (0 = first‐time, 1 = experienced); country (0 = Italy, 1 = UK); woman's age; woman's level of education (0 = lower than university degree, 1 = university degree or higher). Hierarchical regressions were performed to evaluate the role: of parenting experience alone (Step 1); of parenting experience and country (Step 2); of parenting experience and other possible contributing factors (i.e., country, woman's age, woman's level of education) (Step 3). The Benjamini–Hochberg false discovery rate procedure was used to control for multiple hypothesis testing (Benjamini & Hochberg, 1995).

## 7 RESULTS

### 7.1 Descriptive statistics

Table 1 shows demographic information for the overall sample and for the United Kingdom and Italian samples separately. Both samples were representative in terms of being well‐matched to country statistics about education, age, and first‐time versus experienced parents.

**TABLE 1:** Demographic characteristics for the overall sample and separately by country

			Overall sample (*n* = 550)	UK (*n* = 260)	Italy (*n* = 290)	Difference by country
Age (years)	*M* (*SD*)		26.65 (4.71)	25.35 (4.86)	27.82 (4.25)	t (550, 518) = – 6.36, *p <* .001
Parenting experience	%	First‐time	67.1	61.2	72.4	χ^2^ (550, 1) = 7.87, *p =* .005
		Experienced	32.9	38.8	27.6	
Level of education	%	< University degree	60.9	60.1	61.6	χ^2^ (550, 1) = .133, *p =* .716
		> University degree	39.1	39.9	38.4	
			Overall sample (*n* = 550)	First‐time (*n* = 369)	Experienced (*n* = 181)	Difference by parenting experience
Non‐parental caregiving experience	%	None	28.7	28.2	29.8	χ^2^ (550, 3) = .181, *p =* .981
		Little	26.7	27.1	26.0	
		Less frequently	35.1	35.2	34.8	
		Frequently	9.5	9.5	9.4	

Slightly less than 40% of participants had a university degree and this did not differ by country. Consistent with Eurostat fertility statistics (2020), women in the Italian sample were older and more likely to be expecting their first child. Importantly, women expecting their first child were younger than women who already had at least one child. This held in the overall sample (first‐time M = 26.1 years, SD = 4.55; experienced M = 27.75 years, SD = 4.84; t (550, 548) = 3.87, *p* < .001), in the British sample (first‐time M = 24.35 years, SD = 4.22; experienced M = 26.91 years, SD = 5.38; t (260, 258) = 4.05, *p* < .001) and in the Italian sample (first‐time M = 27.44 years, SD = 4.35; experienced M = 28.80 years, SD = 3.85; t = (290, 288) 2.45, *p* = .015). Table 1 also reports non‐parental caregiving experience for the overall sample and separately for pregnant women expecting their first child and pregnant women who already had at least one child.

### 7.2 Preliminary analyses

Variables were first examined for the presence of outliers and tested for normal distribution of the items (Kurtosis and Asymmetry ranging from −1 to +1). As missing data were missing randomly and the amount of missing data was very small (*n* = .005, 0.5% of all data), we replaced missing values at item level with the mean (Kang, 2013).

#### 7.2.1 Confirmatory Factor Analysis (CFA)

We conducted a CFA on the two‐factor, 30‐item BCQ (13 Attunement items and 17 Structure items, following Winstanley & Gattis, 2013). The two‐factor, 30‐item model did not demonstrate adequate fit (RMSEA = .074, CFI = .686, AIC = 1877.8, BIC = 2149.4). Based on the results of the initial CFA, 12 items with factor loadings of .4 or smaller were dropped (see Table 2) (DeVellis, 2016; van de Schoot et al., 2012). We also examined the Modification Indices to identify items that were highly correlated and adjusted model fit using the modification index and standardized expected parameter change (Whittaker, 2012). After these steps, we conducted a second CFA with the revised model (18 items; 7 for attunement and 11 for structure) (see Table 2). The two‐factor 18‐item model demonstrated adequate fit (RMSEA = .057, CFI = .920, AIC = 443.9, BIC = 637.9).

**TABLE 2:** Factor loadings of the Confirmatory Factor Analyses (CFAs) of the Baby Care Questionnaire

	Item		Loading	
	English	Italian	Initial CFA	Final CFA
ATTUNEMENT				
Sleeping				
1	Some days, babies need more or less sleep than other days	In alcuni giorni i bambini hanno bisogno di dormire di più o di meno che in altri giorni	0,15	Deleted
2	It is important for babies to have physical contact with parents when they wake during the night	I bambini traggono beneficio dal contatto fisico con il/i genitore/i quando si svegliano durante la notte	0,43	0,52
3	When babies cry in the night to check if someone is near, it is best to not react	Quando i bambini piangono durante la notte per controllare se qualcuno è vicino è meglio non reagire	−0,63	−0,62
Feeding				
4	Parents should find a pattern of feeding/eating that suits the baby	Il/I genitore/i dovrebbe/dovrebbero trovare una frequenza per i pasti che sia adatta al loro bambino	0,15	Deleted
5	Baby‐led feeding leads to behavioral and sleep problems	Lasciar scegliere al bambino cosa e quando mangiare porta il bambino stesso a sviluppare problemi comportamentali e di sonno.	−0,33	Deleted
6	Offering milk/food to a baby is a good way to test whether she/he is hungry	Offrire del latte/cibo al bambino è un buon modo per capire se ha fame	0,14	Deleted
7	Babies will eat whenever milk/food is offered even if they are not hungry	I bambini mangerebbero ogni volta che gli si offre del latte/cibo anche se non sono affamati	−0,16	Deleted
Soothing				
8	Parents should delay responding to a crying baby	Il/I genitore/i dovrebbe/dovrebbero temporeggiare prima di rispondere ad un/una bambino/a che sta piangendo	−0,73	−0,73
9	It is good to give a baby time to calm him/herself down and increase this amount of time each week	È una buona idea lasciare che il bambino si calmi da solo per un certo tempo, aumentando questo lasso di tempo ogni settimana	−0,67	−0,68
10	Physical contact such as stroking or rocking helps a baby to be calm	Il contatto fisico come l'accarezzare o il cullare aiutano il bambino ad essere tranquillo	0,38	Deleted
11	The more a baby is held, the more he or she demands attention	Più un bambino viente tenuto in braccio, più richiederà attenzioni	−0,64	−0,66
12	Responding quickly to a crying baby leads to more crying in the long run	Rispondere prontamente ad un bambino che piange porta a piu’ pianti nel lungo periodo	−0,64	−0,66
13	Leaving a baby to cry can cause emotional problems	Lasciar piangere un bambino può causare problemi emotivi	0,64	0,50
STRUCTURE				
Sleeping				
1	Babies can have a good night's sleep regardless of scheduling	I bambini possono avere un buon sonno notturno anche non avendo degli orari prestabiliti	0,52	0,50
2	Strict sleeping routines prevent parents from enjoying their child.	Rigide routine di sonno dei bambini impediscono ai genitori di goderseli	0,42	0,39
3	Sleeping schedules make babies unhappy	Avere degli orari prestabiliti per quando dormire rende i bambini infelici	0,47	0,49
4	It is important to introduce a sleeping schedule as early as possible	È importante introdurre il più presto possibile nella vita dei bambini degli orari prestabiliti per dormire	−0,71	−0,69
5	It is important for babies to sleep in a quiet room	E’ importante per i bambini dormire in una stanza silenziosa	−0,29	Deleted
6	It is important for babies to have a sleeping schedule	E’ importante per i bambini avere degli orari prestabiliti per quando domire	−0,72	−0,69
Feeding				
7	Implementing feeding/eating schedules leads to a calm and content baby	Adottare degli orari prestabiliti per mangiare rende i bambini più calmi e contenti	−0,55	−0,56
8	Feeding/eating routines are easy^a^ to follow	È difficile^a^ seguire delle routine per mangiare	0,38	Deleted
9	One danger of feeding/eating schedules is that babies might not get enough to eat	Seguendo degli orari prestabiliti per mangiare c’è pericolo che il bambino non mangi abbastanza	0,49	0,45
10	Following feeding/eating routines prevents parent(s) from enjoying parenthood to the full	Seguire delle routine per mangiare impedisce ai genitori di godersi appieno il loro ruolo.	0,44	0,40
11	It is important to introduce a feeding/eating schedule as early as possible	È importante introdurre il piu presto possibile degli orari prestabiliti per mangiare	−0,67	−0,73
12	Babies will not follow feeding/eating schedules	I bambini non seguirebbero degli orari stabiliti per mangiare	0,20	Deleted
Soothing				
13	Babies with schedules spend less time crying	I bambini che seguono degli orari prestabiliti piangono per meno tempo	−0,50	−0,44
14	Babies cry no matter what their routines	I bambini piangno indipendentemente dalle loro routine	0,33	Deleted
15	Routines lead to more crying	Adottando delle routine i bambini piangono di più	0,32	Deleted
16	Having a set routine helps an upset baby calm down	Seguire delle routine aiuta un bambino agitato ad essere calmo	−0,56	−0,52
17	Babies with regular schedules cry just as much as babies without regular schedules	I bambini che seguono degli orari prestabiliti piangono tanto quanto i bambini che non li seguono	0,40	Deleted

^a^ Due to researcher error the meaning of one item differed in terms of polarity in English and Italian. We adjusted for this error by reversing the values of the Italian item prior to analyses.

#### 7.2.2 Invariance testing

We next tested configural invariance for this two‐factor 18‐item baseline model across countries. Configural invariance refers to the measurement model being an adequate fit for each group separately (Dimitrov, 2010; Schmitt & Ali, 2015; van de Schoot et al., 2012). Goodness‐of‐fit indices reported in Table 3 indicated that model fit for the two‐factor 18‐item baseline model was acceptable for the Italian sample but slightly below recommended thresholds for the British sample. We did not find configural invariance across the British and Italian samples (*Δχ2* = 414.4*; Δdf =* 161*; p <* .001). We then examined model fit across levels of parenting experience. Goodness‐of‐fit indices reported in Table 3 indicated that when considering levels of parenting experience for British and Italian women together, model fit was acceptable for mothers expecting their first child but slightly below recommended thresholds for experienced mothers. When considering levels of parenting experience for British and Italian women separately, model fit indices were below acceptable levels for all four groups. We did not find configural invariance across levels of parenting experience in the British sample (*Δχ2 =* 241.6*; Δdf =* 161; *p <* .001) or the Italian sample (*Δχ2* = 312.1; *Δdf* = 161; *p* < .001).

**TABLE 3:** Model fit indices for the two‐factor 18‐item model

Sample	CFI	RMSEA
Overall sample	.92	.057
Italian women	.907	.057
British women	.893	.073
Experienced mothers (both countries)	.897	.069
First‐time mothers (both countries)	.924	.052
Experienced mothers (Italian)	.808	.088
First‐time mothers (Italian)	.856	.09
Experienced mothers (British)	.89	.061
First‐time mothers (British)	.881	.071

Because we did not find support for configural invariance, we did not test metric or scalar invariance (Schmitt & Ali, 2015). Although Dimitrov (2010) and van de Schoot et al. (2012) state that researchers should not make comparisons between groups without evidence of invariance, Schmitt and Ali (2015) argued that researchers should consider the practical importance of nonequivalence rather than avoid or ignore comparisons between groups. On this basis we proceeded with planned comparisons, including group comparisons. We note however that group comparisons must be interpreted cautiously considering the lack of support for configural invariance.

#### 7.2.3 Scoring and correlations

We calculated scores for Attunement and Structure based on the two‐factor 18‐item baseline model. Table 4 shows the means and standard deviations for Attunement and Structure for the overall sample as well as separately for each country and level of parenting experience.

**TABLE 4:** Descriptive statistics for attunement and structure by country and level of experience

	Attunement			Structure		
	Both countries (*n* = 550)	UK (*n* = 260)	Italy (*n* = 290)	Both countries (*n* = 550)	UK (*n* = 260)	Italy (*n* = 290)
	*M (SD)*	*M (SD)*	*M (SD)*	*M (SD)*	*M (SD)*	*M (SD)*
First‐time	2.67 (.37)	2.62 (.37)	2.71 (.37)	2.84 (.37)	2.76 (.41)	2.90 (.32)
Experienced	2.92 (.39)	2.87 (.40)	2.96 (.37)	2.70 (.46)	2.59 (.49)	2.84 (.37)
Both levels of experience	2.75 (.39)	2.71 (.40)	2.78 (.39)	2.79 (.40)	2.69 (.45)	2.89 (.33)

**FIGURE 1:**
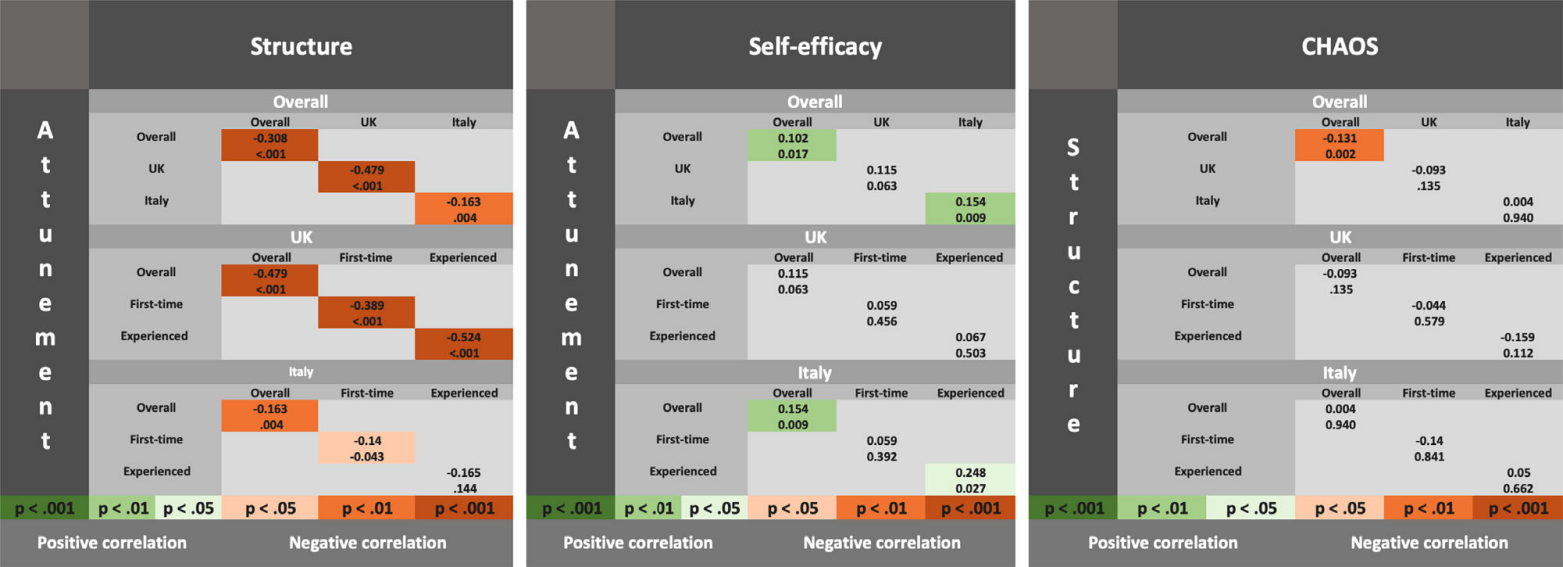
Correlations between attunement, structure, self‐efficacy, and CHAOS

Figure 1 reports Pearson's correlations (a) in the overall sample, (b) considering British and Italian women separately, and (c) considering parenting experience in the two countries separately. Correlations between attunement and structure differed across the British and Italian samples (*z* = −4.16, *p* < .001). For British women, attunement and structure were negatively correlated, while for Italian women, this correlation was weaker or not observed. To evaluate the concurrent validity of the BCQ, Pearson's correlations examined: (1) relations between attunement (BCQ) and self‐efficacy (Maternal Self‐Efficacy in Nurturing Role Questionnaire; Porter & Hsu, 2003); and (2) relations between structure (BCQ) and household environment (CHAOS; Matheny et al., 1995). As predicted attunement and self‐efficacy were positively correlated in the overall sample. When considering the four samples separately, this relation only held for experienced mothers in Italy. As predicted structure and home chaos were negatively correlated in the overall sample. This relation did not hold when considering the four samples separately.

### 7.3 Main analyses

#### 7.3.1 Analysis of variance

Although the lack of support for invariance necessitates caution in comparing means across groups, based on Schmitt and Ali's (2015) advice, we performed two‐way ANOVAs to examine whether parenting beliefs about attunement and structure differed across country and levels of parenting experience. Because participant age differed across the British and Italian samples, the two models controlled for this variable by using age as a covariate. Simple main effects analyses indicated that attunement differed by country (F (550, 1) = 4.59, *p* = .033) and parenting experience (F (550, 1) = 44.32, *p* < .001). Visual inspection of the means in Table 4 reveals that pregnant women who already had at least one child expressed stronger beliefs in attunement compared to women expecting their first child. There was no interaction between country and parenting experience for attunement (F (550, 1) = .005, *p* = .941). Simple main effects analyses showed structure differed by country (F (550, 1) = 30.92, *p* < .001) and parenting experience (F (550, 1) = 10.85, *p* = .001). Visual inspection of the means in Table 4 indicates that Italian women expressed stronger beliefs in structure compared to British women and women expecting their first child expressed stronger beliefs in structure compared to pregnant women who already had at least one child. There was no interaction between country and parenting experience for structure (F (550, 1) = 2.12, *p* = .146).

#### 7.3.2 Hierarchical regressions

To evaluate potential predictors of attunement, hierarchical regressions were conducted with these independent variables: parenting experience (0 = first‐time, 1 = experienced); country (0 = Italy, 1 = UK); woman's age; woman's level of education (0 = lower than university degree, 1 = university degree or higher). Hierarchical regressions were performed to evaluate the role: of parenting experience alone (Step 1); of parenting experience and country (Step 2); of other possible contributing factors (i.e., country, woman's age, woman's level of education) (Step 3). Parenting experience predicted attunement, such that previous experience of parenting predicted higher attunement (*R^2^* = .078, *F* = 45.95, *p* < .001). Moreover, together with parenting experience, country was a significant predictor in the model. Being Italian was associated with higher attunement (*R^2^* = .091, *F* = 27.45, *p* < .001). Finally, together with parenting experience and country, a higher level of education significantly predicted higher attunement (*R^2^* = .108, *F* = 16.97, *p* < .001). Women's age did not significantly predict attunement. Results are summarized in Table 5.

**TABLE 5:** Summary coefficients of regression models for attunement and structure

		Model 1 attunement		Model 2 structure	
Steps	Predictors	*β*	R2 change	*β*	R2 change
Step 1			.080***		.030***
	Experience	.283***		–.175***	
Step 2			.014**		.048***
	Experience	.299***		–.146***	
	Country	–.121**		–.221***	
Step 3			.020**		.009
	Experience	.305***		–.154***	
	Country	–.121**		–.215***	
	Age	.012		.013	
	Level of education	.137**		−.99*	

***p < .001; **p < .01; *p < .05.

To identify factors that may predict structure, hierarchical regressions were used with these independent variables: parenting experience (0 = first‐time, 1 = experienced); country (0 = Italy, 1 = UK); woman's age; woman's level of education (0 = lower than university degree, 1 = university degree or higher). Hierarchical regressions were performed to evaluate the role: of parenting experience alone (Step 1); of parenting experience and country (Step 2); of other possible contributing factors (i.e., country, woman's age, woman's level of education) (Step 3). Parenting experience predicted structure (*R^2^* = .029, *F* = 16.53, *p* < .001). Having parenting experience predicted lower levels of structure. Together with parenting experience, country was a significant predictor: being Italian was associated with higher levels of structure (*R^2^* = .075, *F* = 22.43, *p* < .001). Finally, together with parenting experience and country, level of education significantly predicted structure (*R^2^* = .080, *F* = 12.52, *p* < .001). Women's age did not significantly predict structure. Results are summarized in Table 5.

## 8 DISCUSSION

Our study focused on pregnancy as a foundational period for evaluating parenting beliefs. Building a better understanding of parenting beliefs during pregnancy and the transition to parenthood is important because beliefs influence parenting behavior and child outcomes (Galbally et al., 2018; Gattis et al., 2022; Teti & Gelfand, 1991; Winstanley & Gattis, 2013). Empirical studies of parenting beliefs during pregnancy are needed because most previous studies of parenting beliefs have been conducted with parents already engaged in the task of parenting (e.g., Haltigan et al., 2012; Morrell, 1999; Thompson et al., 2009). We used the BCQ to measure parenting beliefs because we judged it to be more suitable for pregnant women compared to instruments designed for current parents only (Winstanley & Gattis, 2013). We collected data from a large, representative, and culturally diverse sample of Italian and British women. We did not have a priori hypotheses about similarities or differences across these two countries. To examine the role of parenting experience, we compared the beliefs of women expecting their first child with those of pregnant women who already had at least one child, and thus had parenting experience.

We first evaluated the psychometric properties of the BCQ with pregnant women in our sample. We confirmed the previously reported two‐factor structure of the BCQ (Winstanley & Gattis, 2013; Winstanley et al., 2014). Statements about the value of responding to infant cues (i.e., “*It is important for babies to have physical contact with parents when they wake during the night*”) loaded onto attunement, and statements about regularity and routines (i.e., “*It is important to introduce a sleeping schedule as early as possible*”) loaded onto structure. Defining a baseline model with adequate fit for the overall sample did however involve dropping 12 items from the original BCQ based on item communalities and factor loadings. We then evaluated invariance across countries and levels of parenting experience. We did not find evidence of configural invariance across the British and Italian samples or across levels of parenting experience. We proceeded with group comparisons on the basis of Schmitt and Ali (2015), who argued that concerns about interpretability of group comparisons in the absence of evidence for invariance should be weighed against the practical value of group comparisons and the difficulty of demonstrating invariance. We further evaluated the measurement properties of the BCQ by examining the relations between attunement and structure, between attunement and parenting self‐efficacy, and between structure and home chaos. In our British sample attunement and structure were negatively related, as in previous studies with British samples (Winstanley & Gattis, 2013; Winstanley et al., 2014). In our Italian sample however the relation between attunement and structure was negligible, consistent with Winstanley and Gattis’ (2013) proposal that attunement and structure are not mutually exclusive caregiving types, but orthogonal dimensions along which beliefs might vary independently. We found some evidence for the concurrent validity of attunement and structure as measured by the BCQ. For the overall sample, attunement was positively related to self‐efficacy, while structure was negatively related to home chaos.

Our second objective was to evaluate the role of parenting experience in parenting beliefs during pregnancy. Parenting beliefs differed by parenting experience. Pregnant women who already had at least one other child reported stronger beliefs in attunement compared to women expecting their first child. The opposite was true for beliefs in structure: pregnant women with parenting experience reported lower beliefs in structure compared to women expecting their first child. Furthermore, parenting beliefs were related to education: attunement was positively predicted by a higher level of education, whereas structure was positively predicted by a lower level of education. Importantly, our analyses showed that parenting experience was associated with higher attunement and lower structure also when controlling for country, age, and education.

Our study makes several important contributions to the literature on parenting beliefs. First, our results demonstrate that pregnant women have coherent beliefs about attunement and structure. This finding confirms and extends previous evidence of parenting beliefs about attunement and structure in caregiving during infancy (Winstanley & Gattis, 2013; Winstanley et al., 2014). Second, our study provides further evidence of the construct validity of attunement and structure. Attunement was positively related to parenting self‐efficacy, consistent with previous evidence of positive relations between parenting self‐efficacy and responsiveness (Stifter & Bono, 1998; Teti & Gelfand, 1991; Tucker et al., 1998). Structure was negatively related to CHAOS scores: whereas structure reflects the value of regularity and routines, the CHAOS measure indexes disorganization and a lack of routines (Corapci & Wachs, 2002; Wachs & Evans, 2010). Although the lack of invariance means that we should be cautious about comparisons across groups, our study also contributes initial evidence from a large, representative sample in two different countries that parenting beliefs are related to parenting experience. Our findings that pregnant women who already have at least one child have higher beliefs in attunement and lower beliefs in structure compared to pregnant women expecting their first child are consistent with previous evidence that parenting beliefs about nurturing and responding to infants increase with parenting experience, whereas parenting beliefs about restrictiveness, authoritarianism, and limit‐setting decrease with parenting experience (Kahn et al., 2018; Scott & Hill, 2001; Tikotzsky & Sadeh, 2009). We propose that parenting experience increases parents’ knowledge of infant capabilities and needs and that knowledge in turn shapes parenting beliefs about how to care for infants. Future studies should evaluate knowledge of development as well as parenting beliefs to explore this possibility.

Although further evidence including evidence of invariance across levels of parenting experience is needed to warrant strong conclusions, this initial evidence has the potential to make important contributions to practice and policy. In particular, if the relations between parenting experience and parenting beliefs are confirmed by future studies, parent education programmes aimed at new parents might include self‐evaluation of parenting beliefs and knowledge of infant development, with the aim of helping parents develop their knowledge of infant capabilities and needs and as well as an awareness of how knowledge shapes expectations and beliefs about how to care for infants.

Several limitations on our findings need to be considered. In the process of defining a baseline model with adequate fit, a large number of items were dropped. Dropping items may have narrowed the measurement of attunement and structure, and/or masked true differences across experience or culture. Future studies should evaluate whether the measurement model can be improved, for example by revising dropped items to increase clarity and consistency across languages. As previously noted, we did not find support for configural invariance across language and parenting experience, which weakens the validity of comparisons across these groups. We recognized the need for research on parenting beliefs during pregnancy and for cross‐cultural evidence to properly characterize parenting beliefs, and we evaluated invariance based on current guidance. Further research is needed to more accurately identify potential experiential and cross‐cultural differences in beliefs about attunement and structure. Together these results indicate further development of measurement tools is needed for studying attunement and structure. In addition, although experience predicted attunement and structure in our regression analyses, the effects were modest, and the relation between experience and structure was particularly small. Future studies should consider other potential influences on parenting beliefs about attunement and structure, including parenting goals, parents’ own experiences as children, and related hypotheses about intergenerational transmission of parenting (Barrett & Fleming, 2011; Belsky, 1984; Daggett et al., 2000; Hastings & Grusec, 1998; Tamis‐LeMonda et al., 2008) for a more robust set of predictors. Relatedly, because parenting experience may vary with the number of children and/or age of children, future studies should ask participants for this information. Finally, our study used a cross‐sectional design comparing women who differed in parenting experience. Longitudinal evidence is essential for stronger causal inferences about the role of experience in parenting beliefs and for identifying non‐linear changes in beliefs (e.g., Liew et al., 2018; Tikotzsky & Sadeh, 2009).

This cross‐sectional study constitutes an important first step toward a better understanding of parenting beliefs during pregnancy. The results provide initial evidence of the role of parenting experience, culture, and education in parenting beliefs about attunement and structure in caregiving during infancy. More evaluation of equivalence in measurement tools and longitudinal designs will be valuable and will support the logical next steps for stronger inferences and more insights about how experience shapes parenting beliefs.

## CONFLICT OF INTEREST

The authors declare that they have no conflict of interest.

## Data Availability

Study data are available from https://osf.io/q6wzd/.

## References

[imhj22014-bib-0001] Barrett, J. , & Fleming, A. S. (2011). All mothers are not created equal: Neural and psychobiological perspectives on mothering and the importance of individual differences. Journal of Child Psychology and Psychiatry, 52, 368–397. 10.1111/j.1469-7610.2010.02306.x 20925656

[imhj22014-bib-0002] Belsky, J. (1984). The determinants of parenting: A process model. Child Development, 55, 83–96. 6705636 10.1111/j.1467-8624.1984.tb00275.x

[imhj22014-bib-0003] Benjamini, Y. , & Hochberg, Y. (1995). Controlling the false discovery rate: A practical and powerful approach to multiple testing. Journal of the Royal Statistical Society Series B, 57, 289–300. http://www.jstor.org/stable/2346101

[imhj22014-bib-0004] Bozicevic, L. , De Pascalis, L. , Montirosso, R. , Ferrari, P. F. , Giusti, L. , Cooper, P. J. , & Murray, L. (2021). Sculpting culture: Early maternal responsiveness and child emotion regulation–a UK‐Italy comparison. Journal of Cross‐Cultural Psychology, 52, 22–42.

[imhj22014-bib-0005] Bornstein, M. H. , Putnick, D. L. , Park, Y. , Suwalsky, J. T. D. , & Haynes, O. M. (2017). Human infancy and parenting in global perspective: Specificity. Proceedings of the Royal Society B, 284, 20172168. 29237860 10.1098/rspb.2017.2168 PMC5745417

[imhj22014-bib-0006] Bugental, D. B. , & Johnston, C. (2000). Parental and child cognitions in the context of the family. Annual Review of Psychology, 51, 315–344. 10.1146/annurev.psych.51.1.315 10751974

[imhj22014-bib-0007] Coleman, P. K. , & Karraker, K. H. (1998). Self‐efficacy and parenting quality: Findings and future applications. Developmental Review, 18(1), 47–85.

[imhj22014-bib-0008] Colicchia, L. C. , Parviainen, K. , & Chang, J. C. (2016). Social contributors to glycemic control in gestational diabetes mellitus. Obstetrics & Gynecology, 128(6), 13331339. 10.1097/AOG.0000000000001740 27824747

[imhj22014-bib-0009] Corapci, F. , & Wachs, T. D. (2002). Does parental mood or efficacy mediate the influence of environmental chaos upon parenting behavior? Merrill‐Palmer Quarterly, 48(2), 182–201.

[imhj22014-bib-0010] Daggett, J. , O'Brien, M. , Zanolli, K. , & Peyton, V. (2000). Parents’ attitudes about children: Associations with parental life histories and child‐rearing quality. Journal of Family Psychology, 14, 187–199. 10870289 10.1037//0893-3200.14.2.187

[imhj22014-bib-0011] DeVellis, R. F. (2016). Scale development: Theory and applications (4th ed.). Sage.

[imhj22014-bib-0012] Dimitrov, D. M. (2010). Testing for factorial invariance in the context of construct validation. Measurement and Evaluation in Counseling and Development, 43(2), 121149. 10.1177/0748175610373459

[imhj22014-bib-0013] Erkut, S. (2010). Developing multiple language versions of instruments for intercultural research. Child Development Perspectives, 4, 19–24. 21423824 10.1111/j.1750-8606.2009.00111.x PMC3060794

[imhj22014-bib-0014] Eurostat . (2020). Fertility statistics . Retrieved from https://ec.europa.eu/eurostat/statistics‐explained/index.php/Fertility_statistics

[imhj22014-bib-0015] Galbally, M. , Watson, S. J. , Teti, D. , & Lewis, A. J. (2018). Perinatal maternal depression, antidepressant use and infant sleep outcomes: Exploring cross‐lagged associations in a pregnancy cohort study. Journal of Affective Disorders, 238, 218–225. 29886202 10.1016/j.jad.2018.05.025

[imhj22014-bib-0016] Gattis, M. , Au, T. K. F. , Deeg, S. , & Hung, S. (2019). Using cognitive interviews to inform cross‐cultural research on parenting. [Unpublished manuscript]. Cardiff University.

[imhj22014-bib-0017] Gattis, M. , Winstanley, A. , & Bristow, F. (2022). Parenting beliefs about attunement and structure are related to observed parenting behaviours. Cogent Psychology, 9(1), 2082675. 10.1080/23311908.2022.2082675 PMC9844204 36686722

[imhj22014-bib-0018] Goodnow, J. J. (1988). Parents' ideas, actions, and feelings: Models and methods from developmental and social psychology. Child Development, 59, 286–320.

[imhj22014-bib-0019] Haltigan, J. D. , Leerkes, E. M. , Burney, R. V. , O'Brien, M. , Supple, A. J. , & Calkins, S. D. (2012). The infant crying questionnaire: Initial factor structure and validation. Infant Behavior & Development, 35, 876–883. 23007097 10.1016/j.infbeh.2012.06.001 PMC3494785

[imhj22014-bib-0020] Hastings, P. D. , & Grusec, J. E. (1998). Parenting goals as organizers of responses to parent‐child disagreement. Developmental Psychology, 34, 465–479. 9597357 10.1037//0012-1649.34.3.465

[imhj22014-bib-0222] Hughes, S. O. , Cross, M. B. , Hennessy, E. , Tovar, A. , Economos, C. D. , & Power, T. G. (2012). Caregiver’s feeding styles questionnaire. Establishing cutoff points. Appetite, 58, 393–395. 22119478 10.1016/j.appet.2011.11.011 PMC3268070

[imhj22014-bib-0021] Hsu, H. C. , & Lavelli, M. (2005). Perceived and observed parenting behavior in American and Italian first‐time mothers across the first 3 months. Infant Behavior & Development, 28, 503–519.

[imhj22014-bib-0022] Irwin, J. R. (2003). Parent and nonparent perception of the multimodal infant cry. Infancy, 4, 503–516.

[imhj22014-bib-0023] Jansen, E. , Mallan, K. M. , Byrne, R. , Daniels, L. A. , & Nicholson, J. M. (2015). Breastfeeding duration and authoritative feeding practices in first‐time mothers. Journal of Human Lactation, 32, 498–506. 26634270 10.1177/0890334415618669

[imhj22014-bib-0024] Kahn, M. , Bauminger, Y. , Volkovich, E. , Meiri, G. , Sadeh, A. , & Tikotzky, L. (2018). Links between infant sleep and parental tolerance for infant crying: Longitudinal assessment from pregnancy through six months postpartum. Sleep Medicine, 50, 72–78. 10.1016/j.sleep.2018.05.014 30015254

[imhj22014-bib-0025] Kang, H. (2013). The prevention and handling of the missing data. Korean Journal of Anesthesiology, 64(5), 402. 10.4097/kjae.2013.64.5.402 23741561 PMC3668100

[imhj22014-bib-0026] Leerkes, E. M. , Parade, S. H. , & Burney, R. V. (2010). Origins of mothers' and fathers' beliefs about infant crying. Journal of Applied Developmental Psychology, 31, 467–474. 21152107 10.1016/j.appdev.2010.09.003 PMC2997690

[imhj22014-bib-0027] Lester, B. M. , & LaGasse, L. L. (2008). Crying (pp. 80–90). Elsevier.

[imhj22014-bib-0028] Liew, J. , Carlo, G. , Streit, C. , & Ispa, J. M. (2018). Parenting beliefs and practices in toddlerhood as precursors to self‐regulatory, psychosocial, and academic outcomes in early and middle childhood in ethnically diverse low‐income families. Social Development, 27, 891–909. 10.1111/sode.12306

[imhj22014-bib-0029] Lozoff, B. , & Brittenham, G. (1979). Infant care: Cache or carry. Journal of Pediatrics, 95, 478–483. 381622 10.1016/s0022-3476(79)80540-5

[imhj22014-bib-0030] Matheny, A. P. , Wachs, T. D. , Ludwig, J. L. , & Phillips, K. (1995). Bringing order out of chaos: Psychometric characteristics of the confusion, hubbub, and order scale. Journal of Applied Developmental Psychology, 16, 429–444.

[imhj22014-bib-0031] Miller, S. A. (1988). Parents’ beliefs about children's cognitive development. Child Development, 59, 259–285.

[imhj22014-bib-0032] Mindell, J. A. , Sadeh, A. , Wiegand, B. , How, T. H. , & Goh, D. Y. T. (2010). Cross‐cultural differences in infant and toddler sleep. Sleep Medicine, 11, 274–280. 10.1016/j.sleep.2009.04.012 20138578

[imhj22014-bib-0033] Morrell, J. M. B. (1999). The role of maternal cognitions in infant sleep problems as assessed by a new instrument, the maternal cognitions about infant sleep questionnaire. Journal of Child Psychology and Psychiatry, 40, 247–258. 10188707

[imhj22014-bib-0034] Morelli, G. A. , Rogoff, B. , Oppenheim, D. , & Goldsmith, D. (1992). Cultural variations in infants’ sleeping arrangements: Questions of independence. Developmental Psychology, 28, 604–613.

[imhj22014-bib-0035] Palkovitz, R. , & Copes, M. (1988). Changes in attitudes, beliefs and expectations associated with the transition to parenthood. Marriage & Family Review, 12, 183–199.

[imhj22014-bib-0036] Pena, E. D. (2007). Lost in translation: Methodological considerations in cross‐cultural research. Child Development, 78, 1255–1264. http://www.jstor.org/stable/4620701 17650137 10.1111/j.1467-8624.2007.01064.x

[imhj22014-bib-0037] Porter, C. L. , & Hsu, H. C. (2003). First‐time mothers’ perceptions of efficacy during the transition to motherhood: Links to infant temperament. Journal of Family Psychology, 17, 54–64. 10.1037/0893-3200.17.1.54 12666463

[imhj22014-bib-0038] Prevoo, M. J. L. , & Tamis‐LeMonda, C. S. (2017). Parenting and globalization in western countries: Explaining differences in parent–child interactions. Current Opinion in Psychology, 15, 33–39. 28813265 10.1016/j.copsyc.2017.02.003

[imhj22014-bib-0039] Rubin, K. H. , & Chung, O. B. (Eds.) (2006). Parenting beliefs, behaviors, and parent‐child relations: A cross‐cultural perspective. Psychology Press.

[imhj22014-bib-0040] Ryan, R. M. , & Padilla, C. M. (2019). Transition to parenthood. In M. H. Bornstein (Ed.), Handbook of parenting. Vol. 3. Being and becoming a parent (3rd ed., pp. 513–555). Routledge.

[imhj22014-bib-0041] Sadeh, A. , et al. (2016). Low parental tolerance for infant crying: An underlying factor in infant sleep problems? *Journal of Sleep Research*, 25, 501–507. 10.1111/jsr.12401 26990152

[imhj22014-bib-0042] Sadeh, A. , Flint‐Ofir, E. , Tirosh, T. , & Tikotzky, L. (2007). Infant sleep and parental sleep‐related cognitions. Journal of Family Psychology, 21, 74–87. 17371112 10.1037/0893-3200.21.1.74

[imhj22014-bib-0043] Salonen, A. H. , Kaunonen, M. , Astedt‐Kurki, P. , Jarvenpaa, A. L. , Isoaho, H. , & Tarkka, M. T. (2009). Parenting self‐efficacy after childbirth. Journal of Advanced Nursing, 65, 2324–2336. 19761456 10.1111/j.1365-2648.2009.05113.x

[imhj22014-bib-0044] Schaefer, E. S. , & Bell, R. Q. (1958). Development of a parental attitude research instrument. Child Development, 29(3), 339–361. 10.1111/j.1467-8624.1958.tb04891.x 13573503

[imhj22014-bib-0045] Schmitt, N. , & Ali, A. A. (2015). The practical importance of measurement invariance. In C. E. Lance , & R. J. Vandenberg (Eds.), More statistical and methodological myths and urban legends (pp. 327–346). Routledge/Taylor & Francis Group.

[imhj22014-bib-0046] Scott, D. A. , & Hill, J. (2001). Stability and change in parenting beliefs in first‐time mothers from the pre‐ to postnatal period. Journal of Reproductive and Infant Psychology, 19, 105–119. 10.1080/02646830125252

[imhj22014-bib-0047] Sigel, I. E. , & McGillicuddy‐De Lisi, A. V. (2002). Parental beliefs and cognitions: The dynamic belief systems model. In M. H. Bornstein (Ed.), Handbook of parenting: Vol. 3. Status and social conditions of parenting (2nd ed., pp. 485–508). Erlbaum.

[imhj22014-bib-0048] Stifter, C. A. , & Bono, M. A. (1998). The effect of infant colic on maternal self‐perceptions and mother‐infant attachment. Child: Care, Health and Development, 24(5), 339–351. 9728282 10.1046/j.1365-2214.2002.00088.x

[imhj22014-bib-0049] Tamis‐LeMonda, C. S. , Way, N. , Hughes, D. , Yoshikawa, H. , Kalman, R. K. , & Niwa, E. Y. (2008). Parents' goals for children: The dynamic coexistence of individualism and collectivism in cultures and individuals. Social Development, 17(1), 183–209.

[imhj22014-bib-0050] Tucker, S. , Gross, D. , Fogg, L. , Delaney, K. , & Lapporte, R. (1998). The long‐term efficacy of a behavioral parent training intervention for families with 2‐year‐olds. Research in Nursing & Health, 21(3), 199–210. 10.1002/(SICI)1098-240X(199806)21:3<199::AIDNUR3>3.0.CO;2-C 9609505

[imhj22014-bib-0051] Teti, D. M. , & Gelfand, D. M. (1991). Behavioral competence among mothers of infants in the first year: The mediational role of maternal self‐efficacy. Child Development, 62, 918–929. http://www.jstor.org/stable/1131143 1756667 10.1111/j.1467-8624.1991.tb01580.x

[imhj22014-bib-0052] Thompson, A. L. , Mendez, M. A. , Borja, J. B. , Adair, L. S. , Zimmer, C. R. , & Bentley, M. E. (2009). Development and validation of the infant feeding style questionnaire. Appetite, 53, 210–221. 19576254 10.1016/j.appet.2009.06.010 PMC3130353

[imhj22014-bib-0053] Tikotzky, L. , & Sadeh, A. (2009). Maternal sleep‐related cognitions and infant sleep: A longitudinal study from pregnancy through the 1st year. Child Development, 80, 860–874. 19489908 10.1111/j.1467-8624.2009.01302.x

[imhj22014-bib-0054] Valsiner, J. , & Lightfoot, C. (1987). Process structure of parent‐child‐environment relations and the prevention of children's injuries. Journal of Social Issues, 43, 61–72.

[imhj22014-bib-0055] van de Schoot, R. , Lugtig, P. , & Hox, J. (2012). A checklist for testing measurement invariance. European Journal of Developmental Psychology, 9, 486–492. 10.1080/17405629.2012.686740

[imhj22014-bib-0056] van Sleuwen, B. E. , Engelberts, A. C. , Boere‐Boonekamp, M. M. , Kuis, W. , Schulpen, T. W. J. , & L'Hoir, M. P. (2007). Swaddling: A systematic review. Pediatrics, 120, e1097. 10.1542/peds.2006-2083 17908730

[imhj22014-bib-0057] Wachs, T. D. , & Evans, G. W. (2010). Chaos in context. In G. W. Evans , & T. D. Wachs (Eds.), Chaos and its influence on children's development: An ecological perspective (pp. 3–13). American Psychological Association. 10.1037/12057-001

[imhj22014-bib-0058] Weisner, T. S. , & Gallimore, R. (1977). My brother's keeper: Child and sibling caretaking. Current Anthropology, 18, 169–190.

[imhj22014-bib-0059] Whittaker, T. A. (2012). Using the modification index and standardized expected parameter change for model modification. The Journal of Experimental Education, 80, 26–44.

[imhj22014-bib-0060] Willemsen, M. E. , & van de Vijver, F. J. R. (1997). Developmental expectations of Dutch, Turkish‐Dutch, and Zambian mothers: Towards an explanation of cross‐cultural differences. International Journal of Behavioral Development, 21, 837–854. 10.1080/016502597384695

[imhj22014-bib-0061] Willis, G. B. , & Miller, K. (2011). Cross‐cultural cognitive interviewing: Seeking comparability and enhancing understanding. Field Methods, 23, 331–341.

[imhj22014-bib-0062] Winstanley, A. , & Gattis, M. (2013). The baby care questionnaire: A measure of parenting principles and practices during infancy. Infant Behavior & Development, 36, 762–775. 24050932 10.1016/j.infbeh.2013.08.004 PMC3878760

[imhj22014-bib-0063] Winstanley, A. , Sperotto, R. G. , Putnick, D. L. , Cherian, S. , Bornstein, M. H. , & Gattis, M. (2014). Consistency of maternal cognitions and principles across the first five months following preterm and term deliveries. Infant Behavior & Development, 37, 760–771. 25459794 10.1016/j.infbeh.2014.09.005 PMC4266449

[imhj22014-bib-0064] Zeskind, P. S. , & Lester, B. M. (1978). Acoustic features and auditory perceptions of the cries of newborns with prenatal and perinatal complications. Child Development, 49, 580–589. 710187

